# Aging Adults and Seasonal Influenza: Does the Vitamin D Status (H)Arm the Body?

**DOI:** 10.1155/2012/806198

**Published:** 2011-11-15

**Authors:** Pierre Olivier Lang, Dimitrios Samaras

**Affiliations:** ^1^Department of Internal Medicine, Rehabilitation and Geriatrics, Medical School and University Hospitals of Geneva, Hospital of Trois-Chêne, Chemin du Pont-Bochet 3, CH-1226 Thônex-Geneva, Switzerland; ^2^Translational Medicine Research Group, Cranfield Health, Cranfield University, Vincent Building, College Road, Cranfield MK430AL, UK

## Abstract

Vitamin D (VitD), although originally described as an essential hormone for bone and mineral homeostasis, appears to have an active role in regulating specific facets of human immunity. Indeed, VitD has been shown to have significant effects on cytokine production and lymphocyte proliferation. Evidence that VitD affects clearance of selected pathogens is supported by epidemiological and clinical data, while its coadministration with influenza vaccine in mice enhanced both mucosal and systemic antibody responses. This paper aims to examine how VitD may contribute to limiting the burden of influenza infection in the aging and aged adults, a population in which this burden remains considerable. Furthermore, we discuss how VitD status may play a role in host resistance to influenza virus and influence the immunogenicity of the influenza vaccines currently licensed for adults aged 65 years or over by its effects on innate and adaptive immunities.

## 1. Introduction

**Figure 1:**
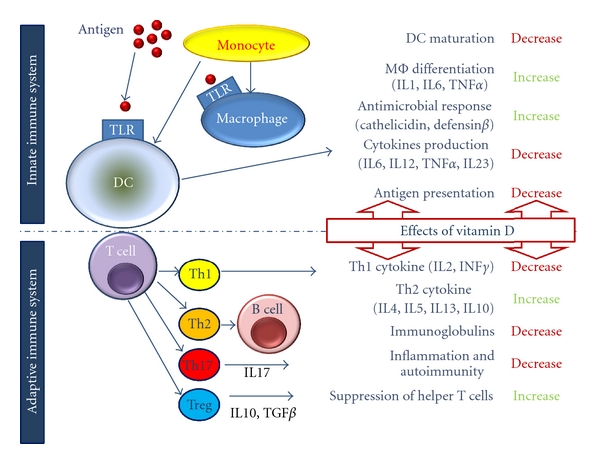
Schematic representation of the immune effects of vitamin D: this figure depicts the principal innate and adaptive immune responses to an antigenic challenge and the influence of vitamin D (positive regulation: increase or negative regulation: decrease) on these responses (B cell: B lymphocyte, cyto T cell, cytotoxic T cell, DC: dendritic cell, MΦ: macrophage T cell: T lymphocyte; TLR: toll-like receptor; TH: helper T cell; Treg: regulatory T cell; IL: interleukin; TNF: tumor necrosis factor; and INF: interferon).

Worldwide, naturally occurring dietary sources of vitamin D (VitD) are limited, and food fortification is often optional, inconsistent, inadequate, or nonexistent [1]. In common with most population subgroups, except infants, adults aged 65 years old or over depend on sunlight for most of their VitD requirements [1, 2]. However, many variables influence the amount of ultraviolet (UV) B (290–315 mm) radiation that reaches the skin and its effectiveness. These include time of day, latitude, altitude, clothing, sunscreen use, pigmentation and age itself [3, 4]. Indeed, even regularly exposed to sunlight, older adults produce 75% less cutaneous VitD than younger adults making them more prone to develop VitD deficiency, defined as a serum or plasma 25-hydroxyvitamin D (25(OH)D) concentration lower than 10 ng/mL (i.e., 25 nmol/L) [4]. In our northern latitudes (including Switzerland), between November and March, there are insufficient UV-B rays to produce VitD. Some reports asserted that 15% of healthier community-dwelling old adults remain VitD insufficient even during summertime, while only 30% reached the desirable plasma levels (>30 ng/mL or 75 nmol/L) at the end of the summer season [5]. Moreover, recent studies have shown that, in the last 10 years alone, serum VitD levels fell on average by 20% [6]. Even though recent evidence suggested that inter-laboratory variability may also contribute to the interpretation of this estimate [7, 8], VitD deficiency is increasingly being recognized as a worldwide epidemic [2, 9, 10]. This statement has led to consider that most of the world's adult population will not be getting an amount of VitD sufficient to maintain healthy bone mass and minimize risk of fracture and of falls [2, 11]. In addition to these usual impacts on bone health, calcium and phosphorus metabolism, decreased VitD status, and/or dietary intakes of VitD have been demonstrated to decrease muscle strength and to increase the risk of type 2 diabetes, atherosclerosis, and neoplastic and immune disorders such as type 1 diabetes mellitus and multiple sclerosis [1, 2, 12]. These findings have led to suggest that VitD could play a role in regulating specific facets of human immunity [12, 13]. This was reinforced by the fact that the VitD receptor (VDR), the receptor that mediates all known vitamin-related biological effects, is widely expressed on cells of the immune system (see Figure 1) [14–16]. This paper will aim to examine how VitD may contribute to limiting the burden of influenza infection in the aged adults, a population in which this burden remains considerable. Furthermore, considerations will be given on how VitD status may play a role in host resistance to influenza virus as well as influence the vaccine immunogenicity and this by its effects on both arms of the immune system (i.e., innate and adaptive immunity). 

## 2. Why Does Seasonal Influenza Infection Remain a Considerable Burden in the Elderly Population?

Worldwide, seasonal influenza infection continues having a considerable impact. Annual estimates indicate that influenza virus causes 3–5 million severe cases resulting in 250,000 to 350,000 deaths worldwide [18, 19]. The highest influenza infection prevalence occurs amongst older adults especially those with chronic medical conditions or immunological disorders. These are causal factors for the increased mortality observed within these high-risk groups [20]. However, mortality is just the tip of the iceberg in terms of disease burden. Recent data demonstrated that influenza can act as a trigger for functional decline leading to disability in some aged individuals and also contributes to excessive hospitalizations, medical visits, and antibiotic prescriptions [21, 22]. Such outcomes represent an additional considerable economic burden amounting to $87 billion each year in the United States [23]. This burden is partly explained by the age-related decline both in nonspecific and specific immune responses leading, respectively, to a weaker host capacity to resist influenza virus and to a less optimal immune response to immunization.

### 2.1. The Nonimmune Specific Mechanisms That Facilitate Influenza Infection

The respiratory tract represents the primary site for the introduction and deposition of potentially pathogenic microorganisms into the body including influenza virus, and this is mainly through inspired air. Defences of the respiratory tract involve both mechanical factors, mucociliary escalator, receptor, and effector molecules of the innate immune system [24]. One of the key players of the defence system is the ciliated epithelium lining the airway which acts through the physical removal both by the ciliary clearance and the cough reflex. In addition, the presence of broad-spectrum antimicrobial agents in the mucus acting as surfactant, the presence of phospholipase A2, the recruitment of phagocytic cells and an inflammatory response all work in collaboration to defend the airways [25]. Although, this sophisticated defence system is weakened with advancing age and even in the absence of overt lung disease [26–31], the intrathoracic dwell time of influenza viruses is increased [29, 32] facilitating any transepithelial migration of the viral particles. Advancing age is, therefore, negatively associated with nasal, large and small airways ciliary clearance. This is due to significantly slower ciliary beat frequency and longer mucociliary clearance time [26, 27]. In addition, mechanical defences are modified by the loss of lung elastic recoil together with increased compliance of the intraparenchymal airway [28]. As demonstrated by experiments on aerosol transmission of influenza virus the tidal breathing volume declines in the aged individuals [31]. Those people often show impaired ventilation function (measured by the FEV1 expressed as a percentage of the volume predicted) which may be due in part to decreased respiratory muscle strength leading to a less forceful coughing [29]. Complementarily, the sensitivity of cough reflex is also decreased throughout all the nasosinusal and tracheobronchial tree [30]. Even if there is no evidence of changes in the cells producing surfactant and modifications of its composition, aged individuals have been found to be subject to a chronic low-grade inflammation which resembles to the one observed in COPD patients. However, similarities are restricted only to qualitative aspects and not quantitative [33]. As the primary contact of pathogens with the adaptive immune system is via the antigen-presenting cells (APCs), the age-related decline in the numbers of these cells contributes to severely impact strategies for coping with influenza. Further impact on the defense against influenza may follow because of alterations in the response to toll-like receptor (TLR) ligands. In a recent study, Panda et al. found substantial decreases in older compared with young individuals concerning TNF-*α*, IL-6, and/or IL-12 (p40) production in myeloid dendritic cells in response to TLR1/2, TLR2/6, TLR3, TLR5, and TLR8 engagement, as well as tumor necrosis factor (TNF)*α* and interferon (IFN)*α* production in plasmacytoid dendritic cells in response to TLR7 and TLR9 engagement [34]. Authors also found higher intracellular cytokine production in the absence of TLR ligand stimulation by APCs in older compared with younger counterparts, suggesting some dysfunction in the regulation of cytokine production. Moreover, they showed a strong association between poor antibody responses to influenza immunization and impaired TLR function in the older individuals. In addition, fever, which is one of the cardinal signs of influenza infection [35], may be absent or blunted 20%–30% of the time in the aged population [36], thus, leading to facilitated virus spreading.

### 2.2. Influenza Vaccine: A Vaccine Less Effective in the Elderly Than Previously Believed

**Figure 2:**
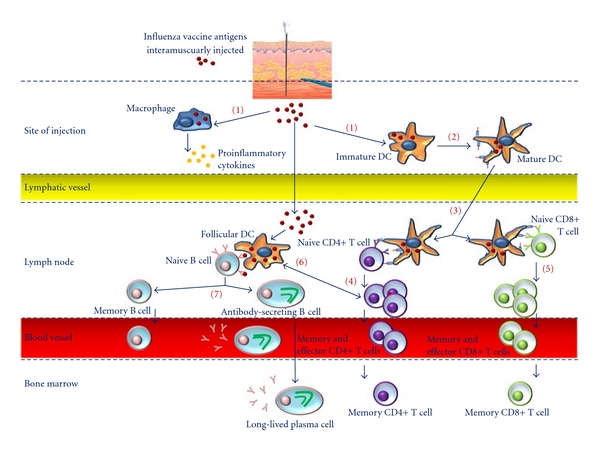
The normal immune response following influenza vaccination. Administration of vaccine antigens induces the activation of the innate immune responses at the site of injection. The antigen is taken up by antigen-presenting cells (1), such as macrophages and dendritic cells (DCs). The local innate immune response facilitates maturation of DCs, which present stable major histocompatibility complex/peptide complexes (2). Mature DCs migrate into lymph nodes (3), where they induce activation and clonal expansion of naive CD4+ (4) and CD8+ (5) T cells. The activation and differentiation of naive B cells is induced by antigen and CD4+ T helper cells (6). Naive B cells differentiate into memory B cells and antibody-secreting B cells (7). Long-term immunity is assured by memory B and T cells in the blood and lymph nodes, as well as by long-lived plasma cells and memory T cells in the bone marrow (adapted from [17]).

The current trivalent inactivated influenza vaccines (TIVs) contain 15 *μ*g of hemagglutinin (HA) of each of the three strains (A/H1N1, A/H3N2, and influenza B), stimulating a response in both B and Tcells, resulting in humoral and cell-mediated immunity, respectively [17, 37]. As depicted by Figure 2, vaccine antigens are taken up by APCs, such as macrophages and dendritic cells (DCs). The local innate immune response facilitates maturation of DCs, which present stable major histocompatibility complex/peptide complexes. Mature DCs migrate into regional lymph nodes, where they induce activation and clonal expansion of naive CD4+ T-helper and CD8+ cytotoxic T cells. The activation and differentiation of naive B cells is induced by antigen and CD4+ T-helper cells. Naive B cells differentiate into memory B cells and antibody-secreting B cells. Long-term immunity is assured by memory B and T cells in the blood and lymph nodes, as well as by long-lived plasma cells and memory T cells in the bone marrow. TIVs are considered as both effective and cost saving in preventing influenza infection in the aged population [18, 19]. However, in spite of widespread application of immunization programmes, rates of hospitalization for acute respiratory illness and cardiovascular disease have been increased in population aged over 65 years during the annual influenza seasons [18, 19]. Thus, vaccine effectiveness, defined as the reduction in attack rates between vaccinated and unvaccinated population, expected to be between 70 and 90% in younger adults is considerably reduced to less than 40% over the age of 65 years [19]. This inability of the immune system to amount an effective response results from a multitude of changes occurring in the immune system when we age. These changes are evident both in the innate immunity and in the adaptive immune system [38, 39]. The cumulative of a life-long reshaping and adaptation of the immune system, in response to exposure to a plethora of pathogenic challenges [40], cellular and molecular changes [41–43], as well as thymic involution [44], is believed to result in this dysfunctional immunity. However, failure of the immune system to provide protection to the body faced with influenza antigen is not only related to immunosenescence [38], but factors such as chronic comorbid conditions [45, 46], nutritional status [47, 48], and hormonal pathway dysregulation [49, 50] seem to be important contributory factors as well [46]. 

## 3. Is VitD a Potential Immune-Enhancing Agent against Influenza?

In order to enhance the immune response and make the ageing body more prone to protect itself from influenza virus, novel vaccine designs and immunological therapeutic approaches have been developed [19]. Whether modestly increased immunogenicity was measured when administrating new adjuvanted, intradermal, or high-dose vaccines, slightly higher side effects have led to lower acceptability [51]. Strategies reaching the goal of rejuvenating the immune system and usually mentioned as the 3R's of rejuvenation (restoration, replacement, and reprogramming) [43] have been mainly achieved in experimental systems. However, no clinical translation of these results is currently available [52], except strategies using nutrients and protein-energy supplements that included VitD as well [12, 53, 54]. 

### 3.1. Does VitD Have an Anti-Infectious Effect?

Recently, there has been a great deal of interest in the role played by VitD in host resistance to infection, and a number of scientific claims reported broad anti-infectious effects, potentially beneficial in people infected with influenza [13, 55, 56]. This emerging role of VitD in innate immunity results in two findings: the immune system is able to produce 1*α*-hydroxylase, the enzyme that converts circulating VitD to its active form [57–60] and the active VitD produced in the immune system led to the production and regulation of antimicrobial peptides such as *cathelicidin* and *defensin *β*2 *[13, 61] which in turn inhibited replication of *Mycobacterium tuberculosis*,* in vitro* [62]. However, recent randomized controlled trials using VitD supplementation (3 doses of 100.000 UI) showed no beneficial effect in clinical outcomes or mortality in tuberculosis [63] while a meta-analysis demonstrated a positive association between VDR polymorphisms with risk of tuberculosis [64]. Even though the latter supported the hypothesis that VitD deficiency might play a role as a risk factor during the development of tuberculosis, another report in dialysis patients showed no association between VitD supplementation and decreased risk of the infection [65]. Evidence that VitD affects clearance of selected infections is also supported by epidemiological data showing an inverted association between serum 25(OH)D level and upper respiratory tract infection incidence rates [6, 66]. This effect was attributed to VitD regulation of antimicrobial peptides such as cathelicidin [61]. In humans, the only known cathelicidin is hCAP-18. It enhances microbial killing in phagocytic vacuoles, acts as chemoattractant for neutrophils and monocytes, and has a defined VitD-dependent mechanism [67, 68]. As depicted by Figure 1, pathogenic antigens interact with TLRs on macrophages to upregulate the expression of genes that code for the VDR and the 1*α*-hydroxylase enzyme [62, 69, 70]. In turn, the biologically active 1,25(OH)_2_D enhances cathelicidin synthesis by interacting with the promoter of the hCAP-18 [71–73]. However, in order to activate hCAP-18 and enhance macrophage function, it is necessary to reach sufficient levels of the circulating form of VitD [6, 62, 73]. Thus, serum 25(OH)D level of 30 ng/mL (i.e., 75 nmol/L) or more is necessary for the optimal induction of cathelicidin messenger RNA. Interestingly, higher levels (40 ng/mL or 100 nmol/L) do not provide additional benefit [6]. Thus, some hypothesized that wintertime VitD insufficiency may explain seasonal variation in influenza infection [61]. Whilst many reports demonstrated the effect of vitamin D on the increased expression of antimicrobial peptides [13, 61, 74], the effect of either VitD or 1,25(OH)_2_D on these specific peptides against influenza infection has not been tested *in vitro* nor *in vivo* [13]. All together these findings demonstrate that it is still difficult to predict what the exact effect of VitD would be on host resistance to influenza virus.

### 3.2. How Could Vitamin D Modulate Influenza Vaccine Immunogenicity?

Recent studies have demonstrated potent effects of VitD on cytokine production and in regulating normal innate and adaptive immune functions in animals and humans [13–16]. Indeed, as shown in Figure 1, the immunomodulatory effects of VitD are thought to be mediated via (i) its action on APCs with the most potent reported effects on DCs [75–77], (ii) T-cell proliferation and cytokine production with inhibition of the Th1-like cytokines (interleukin (IL)2, IFN*γ*), increase of the Th2-like cytokines (IL4, IL5, IL10 and IL13) and of the Th17-like cytokine (IL17) [78, 79], and (iii) through direct effects on B-cell homeostasis, proliferation, and immunoglobulin production [80, 81]. Potential effects on a fourth group of CD4+ T cells exerting suppressor rather than effector functions and known as regulatory T cells or Tregs have been also suspected [82–85]. As a result, VitD coadministrated with TIV in mice was shown to enhance the anti-HA antibody response and mucosal immunity [86]. In humans, no significant difference in HA inhibition titers was detected in a randomized controlled study conducted in healthy volunteers ranging in age from 18 to 49 years, vaccinated with TIV, and randomized for 1,25(OH)_2_D or placebo [54]. Three important limitations to the conclusion are, however, drawn from this study. First, the subjects in this study were young and healthy, and, because their serum 25(OH)D level at baseline was not measured, we can easily consider that these individuals were not VitD deficient. Second, significant prevaccination HA titers were measured in nearly all subjects. This indicates that the subject had considerable immunity to the three vaccine influenza strains before their vaccination. Since a clear inverse relationship exists between preimmunization serum HA antibody levels and antibody increase after vaccination, this could have masked the potential immunomodulatory effect of VitD [19]. Third, vitamin supplementation and vaccine were simultaneously injected. Even if the study used the active form of VitD, the delay between the two injections was too limited to expect any beneficial biological effects on B and T cells or on components of innate immunity. More recently, Chadha et al. investigated the influence of baseline serum 25(OH)D level on serological response to TIV vaccination [53]. While the results demonstrated that there was a significant effect of baseline 25(OH)D when tested as a continuous variable in relation to serological response, the study conclusions were also affected by three important limitations. First, subjects enrolled in the study were only men with cancer, a comorbid condition well known by itself as limiting immune system capacities. Second, at baseline a large number of patients were taking high dose of VitD with a median dose of 2000 IU/day (dose range from 800 to 9.000 UI). Enrolled subjects had median 25(OH)D level of 44.88 ng/mL (i.e., 112.2 nmol/L), much higher than values usually found in most cancer patients population (i.e., 22-23 ng/mL or 55–58 nmol/L) [87]. Third, the small sample size of 60 subjects has probably underpowered the study design. While the beneficial effect observed only with the A/H3N2 strain correlated with previous findings from a randomized controlled trial of VitD supplementation versus placebo in school children [88]; responses to individual strains of influenza vaccine among patients with lowest and highest quartile of 25(OH)D level were not different for A/H1N1 and B strains. Also, when any virus strains were considered, the difference between the vaccine response rates were insignificant, despite a 30% difference between the two groups.

Finally, similar to potential antimicrobial effects of VitD, more evidence is still needed in humans in order to determine if there is a true causal link between changes in VitD and immune system regulation. Continued evaluation of the consequences of Vit D insufficiency appears warranted. Future studies should address the question whether VitD supplementation enhances immune response to influenza vaccination in nonsupplemented population with low baseline serum 25(OH) levels and thus preventing influenza clinical outcomes.

## 4. Conclusion

Influenza infection remains a major public health concern across the world. The overall body of evidence suggests that older adults are more prone to be infected by influenza virus. While influenza prevention strategies are mainly based on immunization, current influenza vaccines do not offer optimal protection in this population due, in part, to waning immunity. Even if VitD has profound effects on immunity and clinical and epidemiological data suggest that VitD insufficiency increases susceptibility to influenza infection, there is not yet sufficient information to clarify the true relationship between VitD status and host resistance or influenza vaccine immunogenicity. It is, therefore, premature at this time to recommend “booster” VitD supplementations at the beginning of annual influenza seasons in order to prevent infection in the elderly population. However, assessing VitD status and maintaining optimal serum levels should be considered in all ageing and old adults in order to prevent bone and promote healthy ageing.
